# Comparative transcriptome analysis of tree *Eucalyptus* species using RNAseq technology: analysis of genes interfering in wood quality aspects

**DOI:** 10.1186/1753-6561-5-S7-P175

**Published:** 2011-09-13

**Authors:** MM Salazar, LC Nascimento, ELO Camargo, RO Vidal, J Lepikson-Neto, DC Goncalves, WL Marques, PJSL Teixeira, GAG Pereira

**Affiliations:** 1 Laboratório de Genômica e Expressão - LGE- UNICAMP, Brazil; 2 Laboratório Nacional de Biociências – CNPEM/ABTLuS, Brazil

## Background

The *Eucalyptus* wood is one of the most important raw materials for pulp and paper industry. Brazil is currently the first producer of short-fiber pulp and sixth in total production of cellulose. To maintain the industrial competitiveness, investment in genomic research started in 2002 with the *GENOLYPTUS* project (Brazilian Network of Eucalyptus Genome Research). Recently, a new transcriptome library was generated using Next Generation RNA Sequencing by Illumina’s sequencing by synthesis technology.

Different species of *Eucalyptus* are recognized for their superior characteristics in terms of growth, wood quality and resistance to different types of stress (1). Such features are probably driven by the coordinated expression of numerous genes involved in processes of structural and regulatory genes in xylogenesis. Therefore, the main purpose of this study is to identify genes and key metabolic compounds directly involved in wood quality, as well as transcription factors involved. An extensive data mining in the RNAseq database was conducted to identify sequences over expressed in xylem and those that were differentially expressed between species.

## Methods

Genolyptus Sanger sequenced ESTs (167,271) and NCBI *Eucalyptus* ESTs (36,981) were assembled using the program CAP3 (2). All unigenes were automatically annotated using BLAST (3) (e-value cutoff of 1e-5) against protein databases, including: non-redundant (NR) database, uniref (4), pfam (5) and keg (6). Moreover, a functional annotation using the BLAST2GO software was performed (7). The RNA-Seq reads produced from three different xylem libraries (*Eucalyptus globulus*, *E. grandis* and *E. urophylla*) were aligned against the assembled unigenes using the SOAP2 aligner (8) configured to allow up two mismatches, discard sequences with “N”s and return all optimal alignments. In order to perform the differential expression analysis between libraries, a normalization and statiscal pipeline were applied using DEG-seq (9) software considering a 99% confidence rate (cut-off of 0.01). From this analysis we obtained xylem genes and transcription factors differentially expressed between the three species.

## Results and discussion

**Figure 1:**
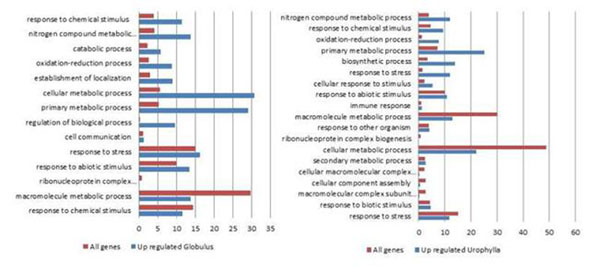
Functional annotation using the BLAST2GO software.

The assembly produced 53,412 unigenes (18,098 contigs and 35,314 singlets). The xylem libraries produced a large number of RNAseq reads (35bp). About 28 million reads were produced for the *E. globulus* library, 25 million for *E. grandis* and 25 million for *E. urophylla.* About 2% of reads were discarded after filtering. Most part of RNAseq reads mapped into the new EST assembly: 69.27% for *E. globulus*, 71.97% for *E. grandis* and 67.90% for *E. urophylla.* As a result, 33,599 unigenes were aligned to the RNAseq libraries. The functional annotations (Figure 1) show percent of genes related to the most relevant GO categories represented in each of the species pairs syudied for Biological Process, level 3.

In the *E. globulu*s X *E. grandis* comparison, most genes are in the macromolecule metabolic process category that includes genes for pectin, cellulose and hemicellulose metabolism and also transcription factors involved in such pathways. Over 10% of these genes are over-expressed in *E. globulus.* Over 30% of the genes are over-expressed in *E.globulus* in the category metabolic cellular process. *In the E. urophylla X E. grandis* comparison, the metabolic cellular process category is representative of the total number of contigs, however, the number of genes over-expressed in *E. urophylla* is much lower. This may be an indicative that genes that participate in such pathways can contribute to the differential wood qualities found in *E. globulus*.

**Figure 2:**
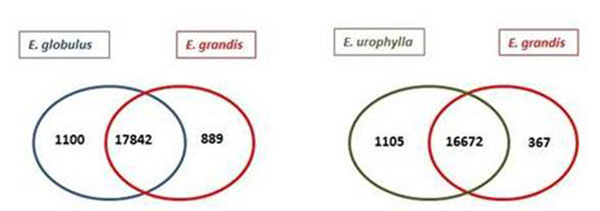
Venn diagrams showing different expression between three distinct xylem libraries.

The new assembly, RNAseq libraries and Gbrowse are available at www.lge.ibi.unicamp.br/eucalyptus. *E. globulus* and *E. urophylla* libraries were compared against *E. grandis* library in order to access differentially expressed genes (considering 99% of confidence rate - cut-off of 0.01). As a result, 19,828 genes were differentially expressed in the *E.gl* X *E. gr* comparison (51.43%) and 18,142 (49.27%) in *E.ur* X *E. gr*. Also in these groups there were genes not expressed in one of the species, as can be seen in Venn diagram below (Figure 2).

These results may contribute to the understanding of wood formation processes and possibly help guide its improvement. The increase in wood quality and productivity has significant economic impacts especially in the pulp and paper industry.
